# Exploring the vector potential of *Lipoptena cervi* (Diptera: Hippoboscidae): first record of *Setaria cervi* (Filarioidea: Onchocercidae) DNA in deer keds

**DOI:** 10.1016/j.crpvbd.2025.100331

**Published:** 2025-10-27

**Authors:** Klaudia Mária Švirlochová, Dana Zubriková, Veronika Blažeková, Lucia Vargová, Eva Čisovská Bazsalovicsová, Ján Čurlík, Ivana Heglasová, Bronislava Víchová

**Affiliations:** a Institute of Parasitology, Slovak Academy of Sciences, Hlinkova 3, 040 01, Košice, Slovakia; b Department of Epizootiology, Parasitology and Protection of One Health, University of Veterinary Medicine and Pharmacy in Košice, Komenského 73, 041 81, Košice, Slovakia; c Department of Breeding and Diseases of Game, Fish and Bees, Ecology and Cynology, University of Veterinary Medicine and Pharmacy in Košice, Komenského 73, 041 81, Košice, Slovakia; d Department of Biology and Environmental Studies, Faculty of Natural Sciences, Matej Bel University, Tajovského 40, 974 01, Banská Bystrica, Slovakia

**Keywords:** *Setaria cervi*, *Lipoptena cervi*, Filarioid nematode, Deer ked, Red deer, Slovakia

## Abstract

*Setaria cervi* is a filarial nematode that infects both wild and domestic ungulates. It is primarily transmitted by mosquitoes, although the possible role of other hematophagous insects is still uncertain. We investigated 83 wingless deer keds (*Lipoptena* spp.) from red and fallow deer in northern and eastern Slovakia, as well as 43 red deer liver samples for the presence of filarial DNA. Deer keds were identified as *Lipoptena cervi* (*n* = 80) and *Lipoptena fortisetosa* (*n* = 3). Genomic DNA from individual ectoparasites was screened by PCR targeting a mitochondrial *cox*1 gene fragment of filaroid nematodes. Two L. *cervi* from red deer in Hrabušice (eastern Slovakia) tested positive for *S. cervi* DNA, with 100% sequence identity with worms recently isolated from Slovak game animals. Additionally, *S. cervi* DNA was detected in one liver from a red deer in the Vranov nad Topl’ou district. This study provides the first molecular evidence of *S. cervi* DNA in *L. cervi*, suggesting a potential role of deer keds in the transmission at the wildlife-livestock-vector interface.

## Highlights

• First detection of *Setaria cervi* DNA in *Lipoptena cervi* deer keds.
• Two L. *cervi* from red deer in eastern Slovakia carried *S. cervi* DNA.
• One red deer liver sample also tested positive for *S. cervi* DNA.
• The findings suggest deer keds may play a role in filarioid transmission.
• The study underscores the need for One Health surveillance of wildlife parasites.

## Graphical abstract

**Figure:**
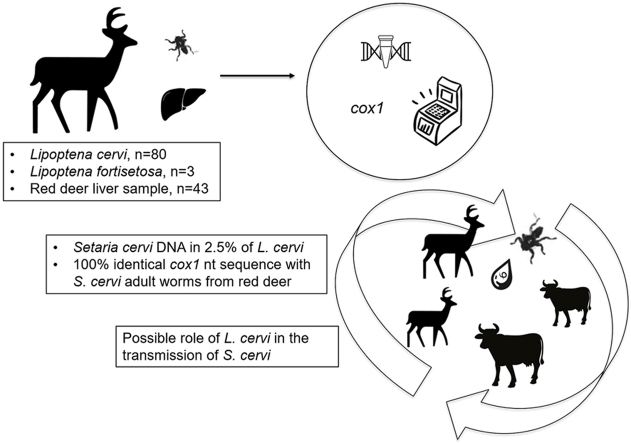

## 1 Introduction

The filarioid nematode *Setaria cervi* (Rudolphi, 1819) (Filarioidea: Onchocercidae) is a vector-borne parasite that commonly parasitizes various ungulate hosts, mainly members of the families Cervidae and Bovidae, residing in the peritoneal or abdominal cavity (Anderson, 2000; Sundar and D’Souza, 2015). Although *S. cervi* is usually found in wild and domestic ruminants, it has also been detected in atypical hosts, such as sheep and goats (Lanková et al., 2021).

In Europe, *S. cervi* was first documented in Germany in red deer (*Cervus elaphus*) (Schwangart, 1940), with subsequent reports confirming its occurrence in red deer, sika deer (*Cervus nippon*), roe deer (*Capreolus capreolus*), and European moose (*Alces alces*) across several central and southern European countries (Blažek et al., 1968; Sugár, 1978; Shimalov and Shimalov, 2003; Rehbein and Visser, 2007; Kuzmina et al., 2010; Alasaad et al., 2012; Angelone-Alasaad et al., 2016; Oloś et al., 2019; Lanková et al., 2021). In Slovakia, *S. cervi* was only recently identified in red deer by Lazár et al. (2024).

Although *Setaria* spp. infections in wild cervids are usually asymptomatic and considered harmless, they can occasionally cause peritonitis, which is potentially life-threatening (Lazár et al., 2024). Additionally, if the parasites reach nervous tissues, they can cause locomotion problems and further paraplegia in the affected animals (Taylor et al., 2015).

*Setaria* spp. are transmitted by the blood-feeding insects, mainly mosquitoes (Diptera: Culicidae) (Oloś et al., 2019). However, blackflies (Diptera: Simuliidae) and some muscid flies (Diptera: Muscidae) have also been proposed as potential vectors (Czajka et al., 2012; Demiaszkiewicz et al., 2015; Kemenesi et al., 2015; Čurlík et al., 2019; Rydzanicz et al., 2019), suggesting that these filarioids may lack strict vector specificity and may also develop in other insect species. Consequently, the broader range of potential vectors could facilitate their wider geographical spread and affect the transmission dynamics.

Infection of the insect vector happens when it ingests first-stage larvae (microfilariae, L1) during a blood meal on an infected host. Inside the insect, L1 larvae develop into the infective third-stage larvae (L3). This development is temperature-dependent and lasts for approximately 14 days (Laaksonen et al., 2009). Transmission to a new mammalian host occurs when L3 larvae are transmitted during the vector’s next blood meal. After transmission, the larvae initially localize in the connective tissue before migrating to the abdominal cavity of the definitive host, where they continue to develop and mature into adult males and females.

Deer keds (*Lipoptena* spp.) are essential ectoparasites of cervids, and in Slovakia, there are two documented species (*L. cervi* and *L. fortisetosa*) (Oboňa et al., 2019, 2022). Infestation levels of *Lipoptena* spp. on individual animals can be very high, with reports of 240 individuals on a red deer, and up to 1400 on a single moose. Co-infestations involving *L. cervi* and *L. fortisetosa* on the same deer host have also been documented (Klepeckienė et al., 2020). Adult deer keds (*L. cervi* and *L. fortisetosa*) are winged when searching for a host but shed their wings permanently upon landing, after which they only occasionally switch hosts (Härkönen et al., 2010; Gałęcki et al., 2021).

In recent years, increasing attention has been directed toward the vector potential of deer keds for bacterial pathogens. These ectoparasites have been shown to harbor a range of bacteria, including *Anaplasma* spp., *Bartonella* spp., *Borrelia* spp., and *Coxiella*-like endosymbionts (Peña-Espinoza et al., 2023; Pearson et al., 2025). Among these, vector competence has so far been experimentally demonstrated only for *Bartonella* spp. (Dehio et al., 2004; Korhonen et al., 2015; Lee et al., 2016). In addition to bacteria, several protozoan pathogens, such as *Theileria luwenshuni*, *T. ovis*, and *Trypanosoma* spp., have also been detected in *Lipoptena* spp. (Böse and Petersen, 1991; Lee et al., 2016; Werszko et al., 2020). In Slovakia, molecular evidence has linked *L. cervi* to *A. phagocytophilum* (Víchová et al., 2011), with additional pathogens potentially involved (Švirlochová et al., unpublished).

Although interest in the ecology and epidemiology of *Lipoptena* spp. is growing, their potential role as vectors of nematodes remains unexplored - even though infestations by deer keds in cervid hosts are typically very high. Given the limited mobility of wingless keds and the restricted location of adult *S. cervi* worms, the transmission of microfilariae by *L. cervi* is likely infrequent, yet still possible, and probably limited to mechanical transfer events. This study aimed to examine the presence of *S. cervi* DNA in *Lipoptena* spp. collected from wild cervids to assess their possible involvement in the transmission cycle of the filarioid nematode.

## 2 Materials and methods

### 2.1 Sampling of deer keds

**Fig. 1:**
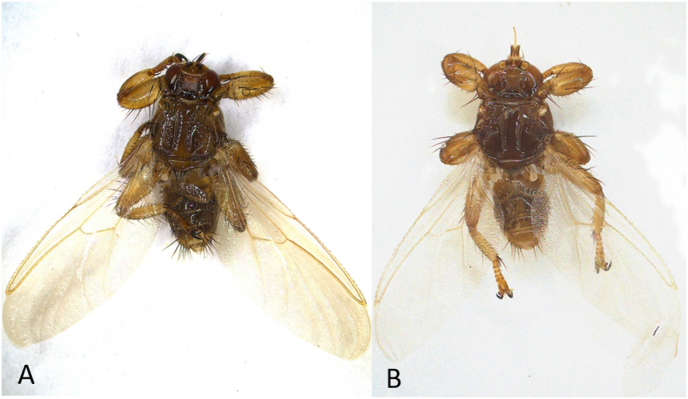
**A***Lipoptena cervi*, dorsal view. **B***Lipoptena fortisetosa*, dorsal view. Images were captured using a stereomicroscope with an Opta-Tech camera and processed in Capture One 2.2.1; final stacking and editing performed in Helicon Focus 8 software. Magnification: 30× .

**Fig. 2:**
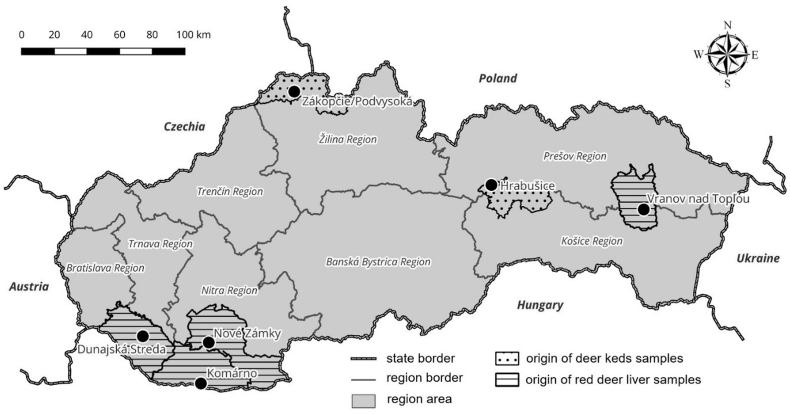
Map of Slovakia with sampling sites.

During September 2024, adult deer keds (Fig. 1) were collected by hunters from the fur of legally hunted fallow deer (*Dama dama*, *n* = 1) and red deer (*Cervus elaphus*, *n* = 6) in Podvysoká/Zákopčie, northern Javorníky Mountains. Additionally, ectoparasites were collected from three red deer (one male and two females) shot during legal hunting in Hrabušice village (Spišská Nová Ves District), on the northern slopes of the Slovak Paradise in eastern Slovakia (Fig. 2). Deer keds were preserved in 70% ethanol and transported to the Institute of Parasitology, Slovak Academy of Sciences (IP SAS). The species and sex of ectoparasites were determined under a stereomicroscope using an identification key (Oboňa et al., 2022).

### 2.2 Animal samples collection

Additionally, genomic DNA was isolated from 43 liver samples from red deer legally hunted in the Vranov nad Topľou District of eastern Slovakia (*n* = 25) and in the southern Slovakia districts (Dunajská Streda, Komárno, Nové Zámky; *n* = 18) of the country (Fig. 2). These tissue samples were provided by hunters some time ago and stored at the IP SAS for further analysis of the presence of vector-borne pathogens. Subsequently, they were included in this study and molecularly screened for the presence of *Setaria* spp. DNA.

### 2.3 DNA extraction and PCR analysis

Before DNA extraction, wingless deer keds were rinsed three times in sterile distilled water for 1–2 min each to remove residual ethanol. The specimens were then placed on filter paper and air-dried for 10–15 min. The whole body of each deer ked was subsequently homogenized individually with a sterile disposable scalpel in a 1.5-ml tube. Genomic DNA was extracted using the NucleoSpin Tissue Kit (Macherey-Nagel, Düren, Germany) following the manufacturer’s instructions, and the extracts were stored at −20 °C until PCR analysis. Total DNA from deer tissue samples was isolated using the same kit and protocol.

All DNA samples (from deer keds and animal tissues) were screened for the presence of filarioid parasites by PCR amplification of a partial (*c.*300 bp long) fragment of the mitochondrial cytochrome *c* oxidase subunit 1 (*cox*1) gene using the primers Cbcox1F (5′-CGG GTC TTT GTT GTT TTT ATT GC-3′) and Cox1intR (5′-ATAAGTACGAGTATCAATATC-3′) designed initially by Casiraghi et al. (2001) and later applied by Latrofa et al. (2012), and Bezerra-Santos et al. (2022).

Amplified PCR products were purified and sequenced bidirectionally using the PCR primers. Nucleotide sequences were analyzed using MEGA 11 software (Tamura et al., 2021) and compared with data available in the GenBank database using Basic Local Alignment Search Tool (BLAST).

## 3 Results

A total of 83 adult wingless deer keds were collected. In particular, 40 deer keds were removed from red deer in Hrabušice and identified as *L. cervi* (10 males and 30 females). Similarly, 40 *L. cervi* (26 males and 14 females) were removed from red and fallow deer shot in Podvysoká/Zákopčie. Additionally, three keds (1 female, and 2 males) removed from hunted red deer were identified as *L. fortisetosa.*

Among the examined DNA samples, two *L. cervi* DNA samples tested positive for the presence of filarioid DNA (2.5%, 2 out of 80 *L. cervi* examined). Both samples originated from red deer shot in Hrabušice in eastern Slovakia. Sequence analyses of the partial *cox*1 (303 bp) gene revealed an identical sequence structure in both analyzed specimens, with no intraspecific variation. Comparison of the obtained sequences with data deposited in the GenBank database revealed 100% sequence identity with the partial *cox*1 of *S. cervi* (GenBank: PP198315, PP198852, PP198854, and PP208633), obtained from the greater omentum of the rumen of red deer from the Slanské Mountains in Slovakia (Lazár et al., 2024). The newly generated sequences of *S. cervi* were deposited in the GenBank database under the accession numbers PV918858 and PV918859.

Overall, *S. cervi* DNA was detected in 1 of 43 red deer liver samples (2.3%). The positive sample originated from the Vranov nad Topľov District. The *S. cervi cox*1 nucleotide sequence (PX063956) obtained from a red deer liver sample was 100% identical with both sequences obtained from deer keds and adult parasites previously isolated from red deer (Lazár et al., 2024).

## 4 Discussion

Deer keds are widely distributed throughout central and eastern Europe, with high prevalences in red, fallow, and roe deer (Kadulski, 1996). In the present study, filarial DNA of *S. cervi* was identified in two specimens of *L. cervi* (prevalence of 2.5%) collected from red deer in Hrabušice, eastern Slovakia. This represents the first molecular evidence of *S. cervi* in deer keds and raises the possibility that *L. cervi* may act either as an incidental host of this filarioid nematode or a mechanical vector. Adult *S. cervi* reside primarily in the peritoneal cavity of deer, while microfilariae are released into the circulating peripheral blood. This blood circulation makes microfilariae accessible to hematophagous ectoparasites and is a key step in the potential transmission of the parasite.

The observed prevalence in *L. cervi* is comparable to that of *Setaria tundra* in mosquitoes in Finland, which ranges from 0.5% to 2.5% (Laaksonen et al., 2009). While the mechanism of transmission remains unconfirmed, our findings add to recent reports of *Setaria* spp. infection in Slovak cervids, including a prevalence of 6.7% in the red deer population (Lazár et al., 2024).

In our study, *S. cervi* DNA was detected in one liver sample (2.3%) from a red deer collected in the Vranov nad Topl’ou District in eastern Slovakia. However, as the liver is not the most suitable tissue for detecting *Setaria* spp. microfilariae, we acknowledge that the prevalence of *Setaria* spp. infection in the examined samples might be significantly underestimated. This finding supports the notion that *S. cervi* is circulating among wild ungulate populations in Slovakia. Nevertheless, to accurately determine the infection rate in game animals from this region, future studies should include screening of blood samples from the specific cervid hosts.

Deer keds are primarily host-specific to cervids, but sporadic infestations in domestic animals (Dehio et al., 2004; Sokół and Gałęcki, 2017) and humans (Härkönen et al., 2009; Kortet et al., 2010; Shimizu et al., 2025) have been documented. The overlapping habitats with livestock and increasing human-wildlife interactions raise concerns about potential interspecific transmission of pathogens, particularly in areas such as forest pastures, where domestic cattle and deer coexist.

Interestingly, in neighbouring Austria, one *L. cervi* individual collected from a red deer featured a *cox*1 sequence of an unknown onchocercid nematode (Filarioidea), most similar (95.1%) to *Mansonella perforata* isolated from Sika deer (*Cervus nippon*) in Japan (GenBank: AM749265). This observation not only raises questions about the presence of *Mansonella* spp. or related species in European deer populations but also highlights the possibility that additional filarioid nematodes may circulate among cervids. Whether this onchocercid sequence truly belongs to *M. perforata* or represents another, previously unsequenced species of *Mansonella* remains to be clarified (Peña-Espinoza et al., 2023).

The findings suggest that *Lipoptena* spp. may play a broader role in the transmission dynamics of filarial nematodes than previously recognized. There is potential for *Lipoptena* spp. to harbour and possibly disseminate a wider diversity of filarial species. Moreover, with the frequent movement of domestic and wild animals across borders, the risk of spreading filarial infections should not be underestimated. While our results do not confirm the vector competence of *L. cervi* for *S. cervi*, they suggest that deer keds may facilitate the ecological maintenance or mechanical transmission of this parasite.

However, as whole-body homogenates of deer keds were used for molecular screening, it remains unclear whether the detected *S. cervi* DNA originated from developing larval stages (L1 to L3) within the insect tissues or merely from ingested microfilariae derived from the blood meal. In mosquitoes, *Setaria* spp. microfilariae migrate to the thoracic muscles, where they develop into infective L3 larvae that subsequently localize in the mouthparts and are transmitted during feeding (Laaksonen et al., 2009). Given the anatomical and behavioral differences between mosquitoes and deer keds, it is uncertain whether *Lipoptena* spp. can support a comparable developmental cycle of filarioid nematodes.

Nevertheless, repeated feeding and close physical contact among cervid hosts increase the likelihood of microfilarial transmission in residual blood meal through contaminated mouthparts or co-feeding. This mechanical route may have epidemiological relevance in densely populated host populations. The present study provides initial evidence linking *L. cervi* to *S. cervi*, alongside the detection of related filarial DNA in red deer in Slovakia, and underscores the importance of investigating deer keds as possible participants in the transmission dynamics of filarioid nematodes.

## 5 Conclusions

Our findings reveal a previously undocumented association between *L. cervi* and *S. cervi*. A combination of microscopic or histological examinations with molecular analyses will be essential to determine whether *L. cervi* serves as a biological vector or represents a mechanical or dead-end host for *S. cervi*. Although the mechanism of transmission remains unclear, this study highlights the importance of continued surveillance for filarial parasites in both arthropod vectors and their wildlife hosts. As interactions between humans, domestic animals, and wildlife intensify, adopting a One Health approach will be crucial for anticipating and mitigating emerging parasitic risks.

## Ethical approval

Not applicable. The wild animals were legally hunted by professional hunters under Slovak legislation. Liver samples were obtained post-mortem in accordance with national ethical standards and legal regulations of the Slovak Republic. All samples were provided to the Institute of Parasitology, Slovak Academy of Sciences, for scientific examination. No animals were killed specifically for this study.

## CRediT authorship contribution statement

**Klaudia Mária Švirlochová:** Investigation, Methodology, Resources, Writing – review & editing. **Dana Zubriková:** Investigation, Methodology, Validation, Writing – original draft. **Veronika Blažeková:** Investigation, Methodology, Writing – review & editing. Lucia Vargová: Investigation, Methodology, Writing – review & editing. **Eva Čisovská Bazsalovicsová:** Funding acquisition, Investigation, Methodology, Project administration, Writing – review & editing. **Ján Čurlík:** Validation, Resources, Writing – review & editing. **Ivana Heglasová:** Investigation, Methodology, Resources. **Bronislava Víchová:** Conceptualization, Data curation, Funding acquisition, Project administration, Supervision, Validation, Visualization, Writing – original draft, Writing – review & editing.

## Funding

This work was supported by the projects APVV-22-0440, 10.13039/501100006109VEGA 2/0033/25, 10.13039/501100006109VEGA 2/0051/24, and SK-10.13039/501100022871SRB 23–0046.

## Declaration of competing interests

The authors declare that they have no known competing financial interests or personal relationships that could have appeared to influence the work reported in this paper.

## Data Availability

The data supporting the conclusions of this article are included within the article. The newly generated sequences were submitted to the GenBank database under the accession numbers PV918858 and PV918859.

## References

[bib1] Alasaad S., Pascucci I., Jowers M.J., Soriguer R.C., Zhu X.-Q., Rossi L. (2012). Phylogenetic study of *Setaria cervi* based on mitochondrial *cox*1 gene sequences. Parasitol. Res..

[bib2] Anderson R.C. (2000).

[bib3] Angelone-Alasaad S., Jowers M.J., Panadero R., Pérez-Creo A., Pajares G., Díez-Baños P. (2016). First report of *Setaria tundra* in roe deer (*Capreolus capreolus*) from the Iberian Peninsula inferred from molecular data: epidemiological implications. Parasites Vectors.

[bib4] Bezerra-Santos M.A., Moroni B., Mendoza-Roldan J.A., Perrucci S., Cavicchio P., Cordon R. (2022). Wild carnivores and *Thelazia callipaeda* zoonotic eyeworms: a focus on wolves. Int. J. Parasitol. Parasites Wildlife.

[bib5] Blažek K., Dyková I., Páv J. (1968). The occurrence and pathogenicity of *Setaria cervi* Rud., in the central nervous system of deer. Folia Parasitol..

[bib6] Böse R., Petersen K. (1991). *Lipoptena cervi* (Diptera), a potential vector of *Megatrypanum* trypanosomes of deer (Cervidae). Parasitol. Res..

[bib7] Casiraghi M., Anderson T.J., Bandi C., Bazzocchi C., Genchi C. (2001). A phylogenetic analysis of filarial nematodes: comparison with the phylogeny of *Wolbachia* endosymbionts. Parasitology.

[bib8] Czajka C., Becker N., Poppert S., Jöst H., Schmidt-Chanasit J., Krüger A. (2012). Molecular detection of *Setaria tundra* (Nematoda: Filarioidea) and an unidentified filarial species in mosquitoes in Germany. Parasites Vectors.

[bib9] Čurlík J., Konjević D., Bujanić M., Sabol Ž., Martinković F., Sindičić M. (2019). The first description of *Setaria tundra* (Issaitshikoff & Rajewskaya, 1928) in roe deer from Croatia. Helminthologia.

[bib10] Dehio C., Sauder U., Hiestand R. (2004). Isolation of *Bartonella schoenbuchensis* from *Lipoptena cervi*, a blood-sucking arthropod causing deer ked dermatitis. J. Clin. Microbiol..

[bib11] Demiaszkiewicz A., Kuligowska I., Pyziel A., Lachowicz J. (2015). First cases of nematodes *Setaria tundra* invasion in elk in Poland. Med. Weter..

[bib12] Gałęcki R., Xuan X., Bakuła T., Jaroszewski J. (2021). Molecular characterization of *Lipoptena fortisetosa* from environmental samples collected in north-eastern Poland. Animals.

[bib13] Härkönen L., Härkönen S., Kaitala A., Kaunisto S., Kortet R., Laaksonen S., Ylönen H. (2010). Predicting range expansion of an ectoparasite – the effect of spring and summer temperatures on deer ked *Lipoptena cervi* (Diptera: Hippoboscidae) performance along a latitudinal gradient. Ecography.

[bib14] Härkönen S., Laine M., Vornanen M., Reunala T. (2009). Deer ked (*Lipoptena cervi*) dermatitis in humans - an increasing nuisance in Finland. Alces.

[bib15] Kadulski (1996). Ectoparasites of Cervidae in north-east Poland. Acta Parasitol. Pol..

[bib16] Kemenesi G., Kurucz K., Kepner A., Dallos B., Oldal M., Herczeg R. (2015). Circulation of *Dirofilaria repens*, *Setaria tundra*, and onchocercidae species in Hungary during the period 2011–2013. Vet. Parasitol..

[bib17] Klepeckienė K., Radzijevskaja J., Ražanskė I., Žukauskienė J., Paulauskas A. (2020). The prevalence, abundance, and molecular characterization of *Lipoptena* deer keds from cervids. J. Vector Ecol..

[bib18] Korhonen E.M., Vera C.P., Pulliainen A.T., Sironen T., Aaltonen K., Kortet R. (2015). Molecular detection of *Bartonella* spp. in deer ked pupae, adult keds and moose blood in Finland. Epidemiol. Infect..

[bib19] Kortet R., Härkönen L., Hokkanen P., Härkönen S., Kaitala A., Kaunisto S. (2010). Experiments on the ectoparasitic deer ked that often attacks humans; preferences for body parts, colour and temperature. Bull. Entomol. Res..

[bib20] Kuzmina T., Kharchenko V., Malega A. (2010). Helminth fauna of roe deer (*Capreolus capreolus*) in Ukraine: biodiversity and parasite community. Vestn. Zool..

[bib21] Laaksonen S., Solismaa M., Kortet R., Kuusela J., Oksanen A. (2009). Vectors and transmission dynamics for *Setaria tundra* (Filarioidea; Onchocercidae), a parasite of reindeer in Finland. Parasites Vectors.

[bib22] Lanková S., Vejl P., Melounová M., Čílová D., Vadlejch J., Miklisová D. (2021). *Setaria cervi* (Filarioidea, Onchocercidae) undressing in ungulates: altered morphology of developmental stages, their molecular detection and complete sequence *cox*1 gene. Parasitology.

[bib23] Latrofa M.S., Dantas-Torres F., Annoscia G., Genchi M., Traversa D., Otranto D. (2012). A duplex real-time polymerase chain reaction assay for the detection of and differentiation between *Dirofilaria immitis* and *Dirofilaria repens* in dogs and mosquitoes. Vet. Parasitol..

[bib24] Lazár J., Šmigová J., Šmiga Ľ., Berrilli F., Lazár P., Čurlík J., Papajová I. (2024). Molecular detection of *Setaria tundra* (Issaitshikoff & Rajewskaya, 1928) and *Setaria cervi* (Rudolphi, 1819) in red deer in Slovakia. Vet. Res. Commun..

[bib25] Lee S.-H., Kim K.-T., Kwon O.-D., Ock Y., Kim T., Choi D., Kwak D. (2016). Novel detection of *Coxiella* spp., *Theileria luwenshuni*, and *T. ovis* endosymbionts in deer keds (*Lipoptena fortisetosa*). PLoS One.

[bib26] Oboňa J., Fogašová K., Fulín M., Greš S., Manko P., Repaský J. (2022). Updated taxonomic keys for European Hippoboscidae (Diptera), and expansion in central Europe of the bird louse fly *Ornithomya comosa* (Austen, 1930) with the first record from Slovakia. ZooKeys.

[bib27] Oboňa J., Sychra O., Greš S., Heřman P., Manko P., Roháček J. (2019). A revised annotated checklist of louse flies (Diptera, Hippoboscidae) from Slovakia. ZooKeys.

[bib28] Oloś G., Nowakowska J., Rojewska S., Welc-Falęciak R. (2019). New findings of *Setaria tundra* and *Setaria cervi* in the red deer (*Cervus elaphus*) in Poland. Parasitology.

[bib29] Pearson P., Xu G., Siegel E.L., Ryan M., Rich C., Feehan M.J.R. (2025). Detection of *Anaplasma phagocytophilum* DNA in deer keds: Massachusetts, USA. Insects.

[bib30] Peña-Espinoza M., Em D., Shahi-Barogh B., Berer D., Duscher G.G., van der Vloedt L. (2023). Molecular pathogen screening of louse flies (Diptera: Hippoboscidae) from domestic and wild ruminants in Austria. Parasites Vectors.

[bib31] Rehbein S., Visser M. (2007). Die Endoparasiten des Sikawildes (*Cervus nippon*) in Österreich. Wien. Klin. Wochenschr..

[bib32] Rydzanicz K., Golab E., Rozej-Bielicka W., Masny A. (2019). Screening of mosquitoes for filarioid helminths in urban areas in south Western Poland - common patterns in European *Setaria tundra* xenomonitoring studies. Parasitol. Res..

[bib33] Shimalov V.V., Shimalov V.T. (2003). Helminth fauna of cervids in Belorussian Polesie. Parasitol. Res..

[bib34] Shimizu K., Shimozuru M., Yamanaka M., Ito G., Nakao R., Tsubota T. (2025). Evaluating the vector potential of deer keds *Lipoptena fortisetosa* for selected pathogens in Hokkaido sika deer (*Cervus nippon yesoensis*). Parasitol. Int..

[bib35] Schwangart (1940). Über die endemische Parese des Rotwildes und Tuberkulose beim Reh. Berl. Münchener Tierärztliche Wochenschr..

[bib36] Sokół R., Gałęcki R. (2017). Prevalence of keds on city dogs in central Poland. Med. Vet. Entomol..

[bib37] Sugár L. (1978). Nematode infection of wild ruminants in Hungary. Parasitol. Hung.

[bib38] Sundar S.T.B., D'Souza P.E. (2015). Morphological characterization of *Setaria* worms collected from cattle. J. Parasit. Dis..

[bib39] Tamura K., Stecher G., Kumar S. (2021). MEGA 11: Molecular Evolutionary Genetics Analysis version 11. Mol. Biol. Evol..

[bib40] Taylor M.A., Coop R.L., Wall R. (2015).

[bib41] Víchová B., Majláthová V., Nováková M., Majláth I., Čurlík J., Bona M. (2011). PCR detection of re-emerging tick-borne pathogen, *Anaplasma phagocytophilum*, in deer ked (*Lipoptena cervi*) a blood-sucking ectoparasite of cervids. Biologia.

[bib42] Werszko J., Steiner-Bogdaszewska Ż., Jeżewski W., Szewczyk T., Kuryło G., Wołkowycki M. (2020). Molecular detection of *Trypanosoma* spp. in *Lipoptena cervi* and *Lipoptena fortisetosa* (Diptera: Hippoboscidae) and their potential role in the transmission of pathogens. Parasitology.

